# Supportive Noninvasive Tool for the Diagnosis of Breast Cancer Using a Thermographic Camera as Sensor

**DOI:** 10.3390/s17030497

**Published:** 2017-03-03

**Authors:** Marco Antonio Garduño-Ramón, Sofia Giovanna Vega-Mancilla, Luis Alberto Morales-Henández, Roque Alfredo Osornio-Rios

**Affiliations:** Facultad de Ingeniería, CA Mecatrónica, Universidad Autónoma de Querétaro, Campus San Juan del Río, Av. Río Moctezuma 249, Col. San Cayetano, C.P. 76807, San Juan del Río, Querétaro, Mexico; mgarduno01@alumnos.uaq.mx (M.A.G.-R.); sofiavema@hotmail.com (S.G.V.-M.); luis.morales@uaq.mx (L.A.M.-H.)

**Keywords:** breast cancer, infrared thermography, automatic segmentation

## Abstract

Breast cancer is the leading disease in incidence and mortality among women in developing countries. The opportune diagnosis of this disease strengthens the survival index. Mammography application is limited by age and periodicity. Temperature is a physical magnitude that can be measured by using multiple sensing techniques. IR (infrared) thermography using commercial cameras is gaining relevance in industrial and medical applications because it is a non-invasive and non-intrusive technology. Asymmetrical temperature in certain human body zones is associated with cancer. In this paper, an IR thermographic sensor is applied for breast cancer detection. This work includes an automatic breast segmentation methodology, to spot the hottest regions in thermograms using the morphological watershed operator to help the experts locate the tumor. A protocol for thermogram acquisition considering the required time to achieve a thermal stabilization is also proposed. Breast thermograms are evaluated as thermal matrices, instead of gray scale or false color images, increasing the certainty of the provided diagnosis. The proposed tool was validated using the Database for Mastology Research and tested in a voluntary group of 454 women of different ages and cancer stages with good results, leading to the possibility of being used as a supportive tool to detect breast cancer and angiogenesis cases.

## 1. Introduction

Breast cancer is the leading disease both in incidence numbers and mortality among women [1]. Although not exclusive to women, the incidence number is one hundred-times higher than in men, representing the first cause of death in the female population in under-developed regions [2]. Such a situation can be attributed to late diagnosis, which occurs when the cancer has spread out or metastasized, causing devastating results; on the other hand, when early diagnosis is performed, the survival index can go up to 95% [3]. There are multiple techniques that aim at an early detection of breast cancer. Among those, mammography, considered as the gold standard method, is the most used tool to detect breast cancer [4]. However, mammography supposes certain disadvantages like being a painful method and the necessity of exposure to ionizing radiation [5]; besides the problems for detecting small-sized tumors in women with dense breast tissue [6]; and finally, in some countries, mammography is only applied to women over 40 years old [7] once or twice a year. Because of this, the use of additional non-invasive sensing technologies help the experts in the detection and diagnosis of breast cancer, which also face the problems presented in mammography, which are very important because, at the end, this provides higher chances of survival [8].

Multiple sensors have been developed that are applied to daily life and in very specific fields. In the literature, the use of sensors for cancer detection and treatment has been well documented; for example, hyperspectral cameras have been used for the detection of gastric cancer [9], as well as the development of a force sensor to minimize the needle deflection in cancer treatment has been documented [10]; besides, sensors can improve existing tools used in cancer treatment, which aim at minimizing pain in treatments [11]. Temperature is an important physical feature whose measurement is commonly used for fault or disease detection in industrial and medical fields. There are multiple techniques used for heat measurement, which include the use of sensors such as thermocouples to analyze stress in diesel motors [12] and to observe the behavior of a tooth exposed to different LED lights [13]; RTD (resistance temperature detector) is applied to support the process realized in solar dryers [14] or in distillation [15], as well as in civil engineering to monitor high temperature environments [16]; pyrometers are used for the control of temperature in spark plasma sintering [17]; coming to implement ultrasonic [18,19] and optical measuring techniques [20] to complement industrial processes in a non-intrusive way. Infrared (IR) sensing is a technology whose importance has grown in the last few years in multiple applications. Infrared thermography is a non-destructive technique that measures the infrared radiation emitted by a body whose temperature is above absolute zero [4]. Some uses of IR thermography in industry and medicine will be further presented. In industrial applications, IR thermography is used to analyze the delamination in composites [21,22] and to perform the automatic detection of defects in materials [23]. This technology ends up being an extraordinary tool to analyze physical bodies, supported, in most of the cases, by image processing techniques applied to the produced thermograms. In medical applications, IR thermography has been widely used; this is because it is a safe, non-invasive and low-cost technique [24]. Some of its applications include: the treatment of diabetes [25], quick detection of seasonal influenza [26], study of eye diseases [27], the analysis of chronic pain [28] and mainly in the diagnosis of one of the most important diseases of recent times, cancer [29]. Cancer diagnosis using IR thermography is possible since there is a relation between an increment of temperature in the skin and the presence of a tumor, as established by [30]. Because of this, by using this sensing technology, the detection of multiple types of cancer, such as melanoma [31], is possible. Specifically, for breast cancer detection, it is said that asymmetrical breast temperature can be indicative both of vascular problems or cancer in such a region [32]. Although in some works, a temperature difference of 2 °C between the cooler and warmer regions and between symmetric areas of the two breasts is considered normal [33], a difference that ranges from 1 °C to 2.5 °C [34] can be considered suspicious. A temperature difference of 1 °C can be used to detect problems such cancer and angiogenesis. Such a range can be used also to detect benign tumors [35]. Most of the reported methodologies to segment and analyze areas of interest in breast thermograms to perform a diagnosis have a manual implication. Some methodologies need an expert that supports the entire process, from the acquisition and processing to the final evaluation and analysis of the thermograms [36,37]. Other works implement image processing algorithms whose purpose is to segment thermograms in a semi-automatic way, that is using the experience of the user, who helps to guide the segmentation task, and from there, the breast thermograms evaluation is performed automatically [38,39]. Methodologies that seek to perform an automatic diagnosis mention the importance of establishing a protocol for thermograms’ acquisition, resulting in being fundamental for the correct work of the implemented image processing algorithms. In computer-aided diagnosis (CAD), it is important to follow protocols, as they allow one to detect problems even in dense breasts, with the development of intelligent applications that focus on specific regions of interest (ROI), which are at the level of manual segmentations [40]. Static and dynamic protocols are the two most used; the first one aims at reaching a thermal stabilization after 10 to 15 min with the patient at rest and then capture a single image; meanwhile, the second protocol, consists of using some means to cool the chest area to a certain temperature and then capture a series of thermograms every 15 s during 5 min [41]. The extraction of texture features and others, like energy, entropy or contrast, from the acquired thermograms, help to discriminate abnormalities, where aspects like high sensibility and specificity are always considered to provide better results to help specialists [42,43]. In conclusion, although some works that focus on performing the detection of breast cancer using IR thermography have been developed, they still present drawbacks like: the permanent necessity of an expert, which is not always possible, e.g., in marginalized areas in developing countries; besides, there is the necessity for methodologies that process thermograms treating them like thermal matrices instead of simple grayscale images, improving the resolution aspect; the core of these methodologies is to analyze the breast region, automatically eliminating the undesirable zones and providing both an evaluation and diagnosis of the information found in the segmented thermograms, with the purpose of exposing the detected abnormalities, in order to help the experts in the future analysis of breast cancer. Finally, it is necessary that these methodologies report in an appropriate way the protocol and setup used for thermograms’ acquisition, which will facilitate the process of replicating them by non-expert people in necessary places.

This paper presents a supportive tool for breast cancer detection using IR thermography as a sensor. The contribution of this work is the proposal of an automatic methodology for breast area segmentation based on the concepts of thresholding, region of interest and morphological algorithms; besides, a protocol and setup for thermograms acquisition are presented, taking into account a specified time to achieve a thermal stabilization to avoid undesirable temperature measurements. The proposed methodology has the advantage of treating the information provided by the IR thermography sensor as a thermal matrix instead of an image in grayscale or false color, which provides a higher certainty in the resolution aspect. An evaluation of the segmented thermograms is performed looking for asymmetrical temperature values, but also, an identification of the hottest point in the breast area is performed trying to serve as a reference for a posterior analysis by an expert. The proposed tool was validated using the Database for Mastology Research (DMR) with a 79.605% of coincidence, although with a significant difference of thermal stabilization of 60%, and also was tested in a sample of 454 voluntary women of different ages, health and in multiple stages of breast cancer; the outcome was evaluated with the help of an oncologist, contrasting the results with previous diagnosis achieved with the help of mammography.

## 2. Materials and Methods

**Figure 1:**
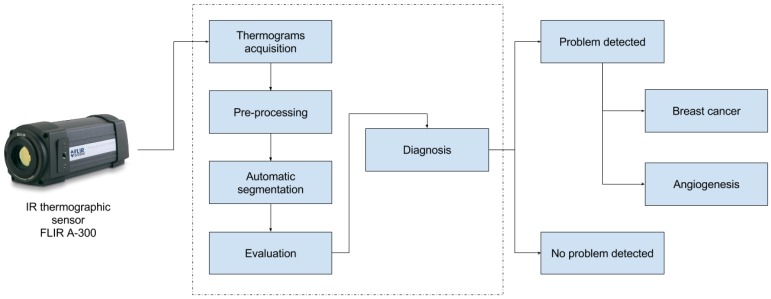
Proposed methodology.

In this section, the proposed methodology to diagnose breast cancer is presented within the theoretical concepts that are used. First, the proposed protocol for the breast thermograms’ acquisition is indicated. Next, the pre-processing stage of the images acquired by the sensor is presented, whose purpose is to facilitate the subsequent steps and eliminate undesired background regions in the thermal images. Then, the automatic segmentation process is shown where the breast region is separated from the patient. At this point, statistical tools are used to evaluate the segmented breast thermograms looking for asymmetrical temperatures. Finally, the diagnosis stage is detailed, the purpose of which is to establish if the patient is healthy or if a problem is detected, searching for the hottest region according to the different temperatures registered by the IR thermography sensor so as to establish the presence of a tumor from cancer or an angiogenesis problem. This workflow can be seen in the Figure 1 in the form of a block diagram, which also serves as a starting point to present the proposed methodology for this paper.

### 2.1. Thermograms’ Acquisition

The starting point for this work consists of establishing a protocol and setup for the thermograms’ acquisition. This depends on the features of the sensor used, and it is expected to take into account the characteristics of the implemented image processing algorithms in order to achieve an efficient breast area segmentation.

#### 2.1.1. Exclusion Criteria

The exclusion criteria define a series of requirements whose target is to increase the possibilities to achieve an appropriate diagnosis. These conditions are highly recommended to be followed as they help to avoid misleading temperatures that result in a false detection. These criteria are the following: (1) avoid the use of: lotions, cosmetic creams, perfumes, deodorant or antiperspirant in the breast area; (2) not to shave the breast area on the test day; (3) not to drink alcohol 24 h before the test day; (4) not to drink caffeinated beverages 24 h before the test day; (5) avoid smoking 2 h before the procedure; and (6) avoid exercise 1 h before the study.

#### 2.1.2. Acquisition Type and Patient Posture

**Figure 2:**
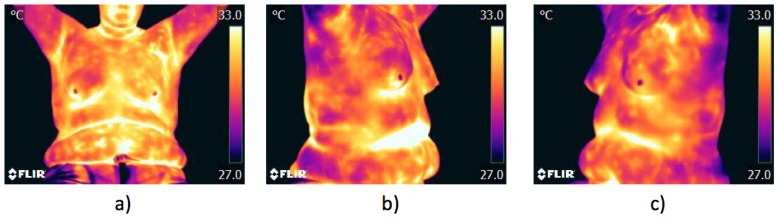
Thermograms acquisition: (**a**) frontal view; (**b**) right view; and (**c**) left view.

The acquisition type for thermograms can be classified into two types: static and dynamic [44]. For the patient posture, although there are multiple works that propose the positions and number of takes [44,45], in this work, the proposal of [46] is used since three images per patient are recommended to be taken. These images include an image from the frontal plane shown in Figure 2a and two semi oblique images, one from the right view and another from the left plane; such images are shown in Figure 2b,c, respectively. This method has the advantage of covering all of the observable breast region; besides, the patient remains seated with the hands in the nape to assure uniform cooling.

#### 2.1.3. Thermal Stability and Emissivity

**Figure 3:**
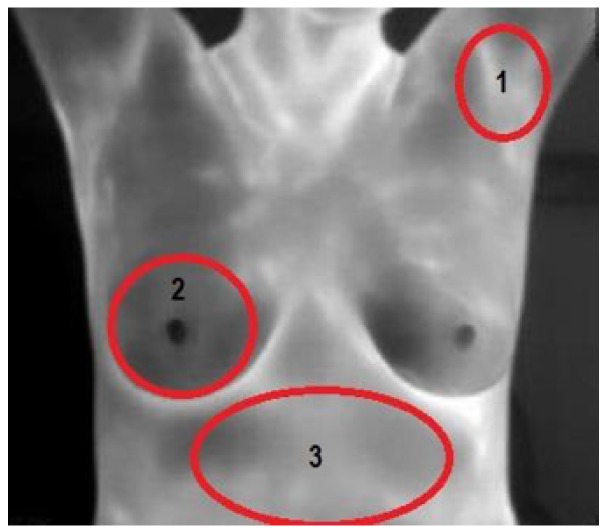
Selected areas to establish the thermal stability.

In order to validate the required time that the patient needs to be in a resting state inside the test place with a controlled ambient environment, a series of periodic measurements were performed. For this, three different body areas were analyzed according to [47]; such areas are shown in Figure 3 and denoted with numbers from 1 to 3, 1 corresponding to the left armpit, 2 to the right breast and 3 to the abdomen region.

**Figure 4:**
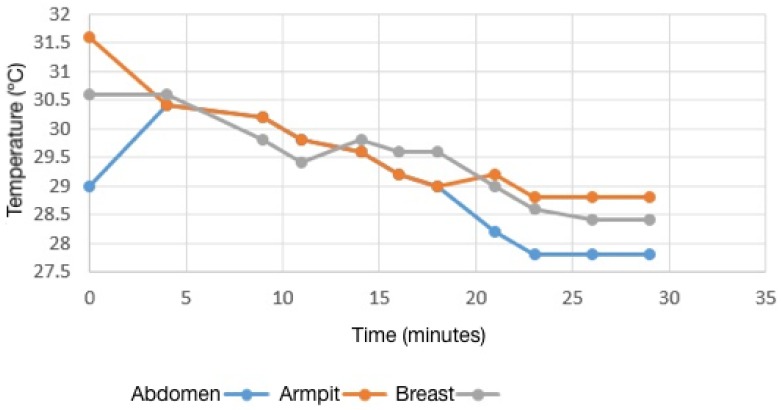
Temperature behavior of selected areas through time.

Areas denoted in Figure 3 were measured during a time interval until such temperatures showed a stable value. Figure 4 shows the decrease of the average temperature value of analyzed regions of 15 different patients. This behavior allows establishing a recommended time of 25 min until an optimal thermal stability is reached prior to performing a diagnosis test.

Finally, there are other factors that influence the resulting acquired thermogram. One of the main ones to take into consideration is the human body emissivity. A value of corporal emissivity of 0.97 is used to consider the measurement results reported by [48].

#### 2.1.4. Sensor

**Figure 5:**
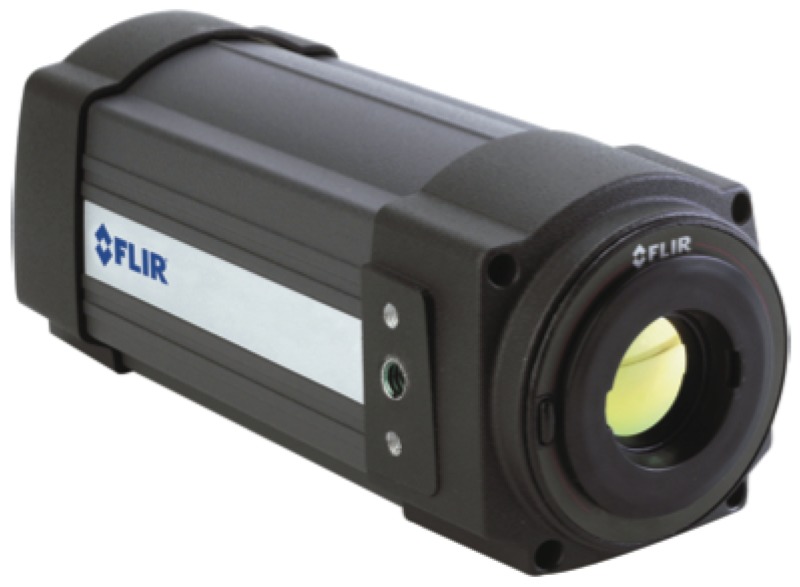
FLIR A-300.

In the present research, the sensor used consisted of an IR thermographic camera FLIR Model A-300, which is shown in Figure 5.

The specific characteristics of the IR thermographic sensor FLIR A-300 are shown in Table 1, where it is important to highlight the sensibility presented by such a device, besides the use of a communication protocol popular in industry and the use of a common image format to represent the acquired thermogram.

**Figure 6:**
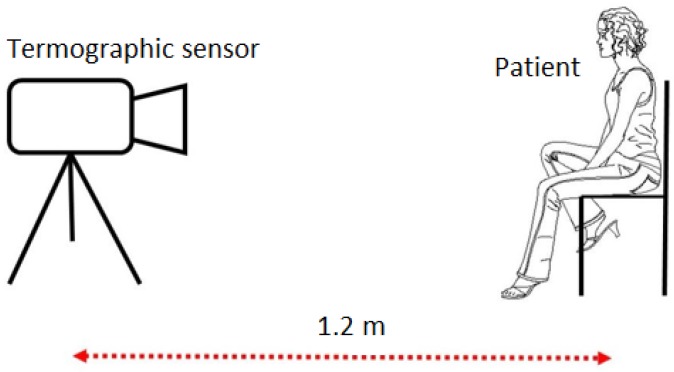
Proposed setup for the thermograms acquisition.

The IR thermographic sensor is placed over a tripod at a distance of 1.2 m from the patient, who is seated in a relaxed position, based on the work distance recommended by [49]. This acquisition setup is shown in Figure 6.

From this value and according to the information of the sensor manufacturer, the theoretical minimum spot size that can be measured is spot size = [IFOV/1000] × distance to target, so in the performed tests at 1.2 m, this value corresponds to 0.001632 m. However, this theoretical value considers perfect lenses, which does not occur in practice. Because of this, a safety margin of 3 should be considered in the term measurement filed of view (MFOV). Considering an MFOV = 3 × IFOV, at 1.2 m of distance, the minimum spot size measurable belongs to 0.5 cm, which allows one to measure cancer tumors from T1b(larger than 0.5 cm) onwards.

### 2.2. Pre-Processing

**Figure 7:**
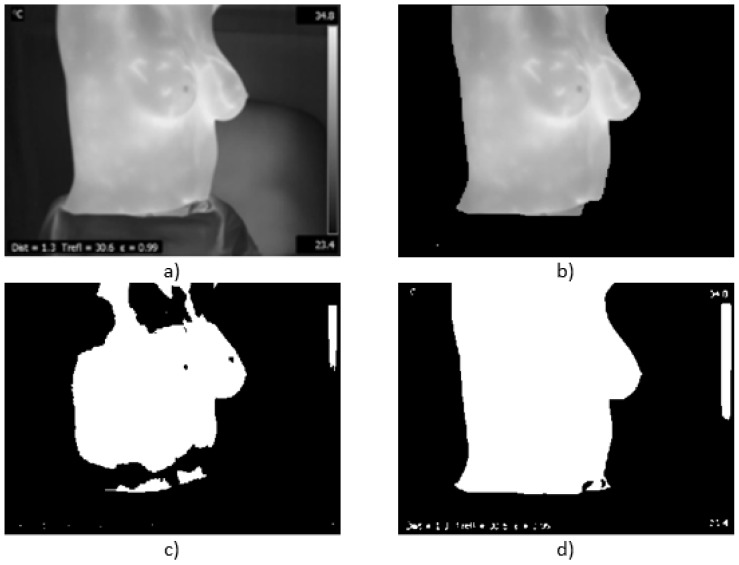
Patient segmentation from the background: (**a**) original thermogram in gray scale; (**b**) GrabCut method; (**c**) Hamadani’s method; and (**d**) Otsu’s method.

The main goal of the pre-processing stage consists of separating the background from the patient in the acquired thermogram. In the literature, there are multiple reported techniques to achieve this, resulting in the threshold being the most used tool. Although the most basic threshold algorithm is a manual technique where the user establishes the intensity level at which the regions should be separated based on its intensity gray level value, automatic techniques have been developed to achieve this based on the image characteristics that works founded on global or local information. Some automatic threshold algorithms include the proposals by Pun, Yen, Kapur and Illingwort; however, one of the most used is Otsu’s method. Otsu’s method can be implemented as stated by [50]. Suppose that the pixels in an image are in *L* gray scale levels in the range [0,L−1]. Otsu established that the optimal threshold $t^{*}$ can be estimated using a discriminating analysis and by maximizing the variance between-class ($\sigma_{B}^{2}$) as shown in Equation (1).

(1) $$t^{*}={ArgMax}_{0\leq t\leq L}\sigma_{B}^{2}\left(t\right)$$

where the between-class variance $\sigma_{B}^{2}$ is defined as denoted by Equation (2).

(2) $$\sigma_{B}^{2}\left(t\right)=w_{1}\left(t\right){\left(\mu_{1}\left(t\right)-\mu_{T}\right)}^{2}+w_{2}\left(t\right){\left(\mu_{2}\left(t\right)-\mu_{T}\right)}^{2}$$

where $w_{1}$ and $w_{2}$ denote the probabilities of the two classes separated by the threshold value *t* and $\mu_{1}$ and $\mu_{2}$ represent the mean gray values of the same two classes. Otsu’s method allows an automatic segmentation of the patient from the background without the need for an additional intervention. This pre-processing step is shown in Figure 7. Figure 7a is the thermogram acquired using the IR sensor with the thermal image in gray scale. Figure 7b shows the segmentation process using GrabCut [51], which behaves excellently when the required marker is selected correctly; meanwhile, in Figure 7c can be seen the result of using Hamadani’s method with $k_{1}$ and $k_{2}$ equal to 1 [52]. Finally, in Figure 7d, the Otsu method’s segmented area is depicted. For the GrabCut case, when the marker is not set correctly or when the object of interest is displaced out of the marker zone, it fails to segment. Otsu’s method showed an adequate behavior for the type of tests performed; the temperature scale indicators being always in the same position are easily trimmed from the image in an additional step.

### 2.3. Automatic Segmentation

**Figure 8:**
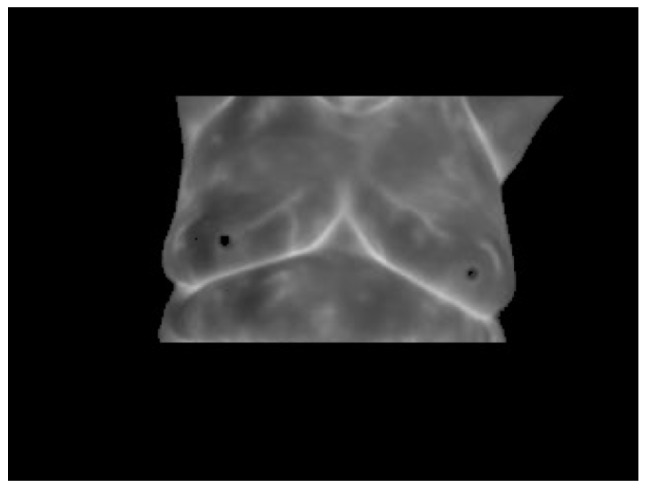
Thermogram acquired at a 1.2-m distance.

Once the patient area has been separated from the background, a segmentation stage is required to extract, from the remaining thermogram, the breast area; then, a comparison between right and left breast should be performed in order to find a significant difference of temperatures. Breast area segmentation can be a challenging task to achieve because of the amorphous nature of such a region. Every person has a different anatomy, making the segmentation task difficult; besides, there is not a physical reference in the body that can be used to ease the process, leading to the use of manual segmentations in multiple works. In this paper, to separate the breast area, some variables can be used because they are under certain control. For example, in the proposed research, the thermograms are acquired at a distance of 1.2 m, while the patient holds a known posture with her hands in the nape, as illustrated in Figure 8.

**Figure 9:**
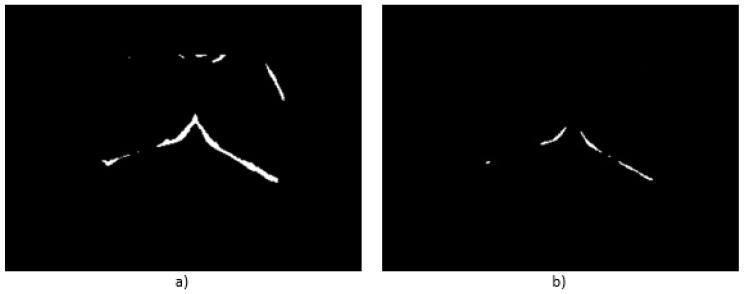
Automatic detection of inferior breast limits: (**a**) regions above the selected threshold value; and (**b**) thicker regions detected using the distance transform.

Another advantage of the proposed protocol for the thermograms’ acquisition is the establishment of the required time to reach the thermal stability. With this, the natural characteristic presented in the area below the breast, which is one of the regions with the highest temperature, can be used to automatically infer its location by using a simple threshold. The value of the threshold is automatically determined considering the highest intensity gray level found in the image, minus 10 (a value found heuristically by various tests). Although, multiple objects can be found in that range, as seen in Figure 9a, the area below the breast also usually presents a thick structure, enough to be marked as the largest object after applying a distance transform. The result of such a process can be seen in Figure 9b, in which the lines that denote the highest points of the brightest (or the darkest, considering the inverted thermogram from Figure 8) and thickets regions that were isolated with the distance transform are shown.

**Figure 10:**
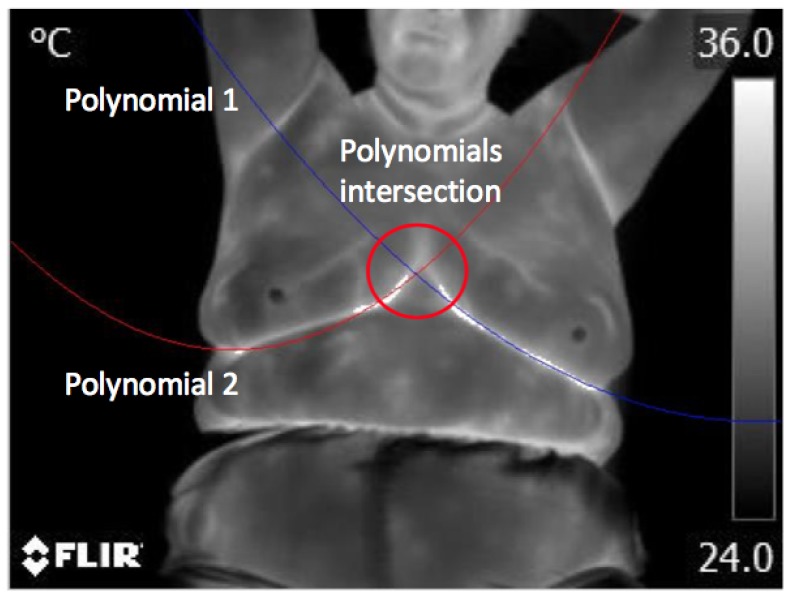
Polynomials to fully find inferior limits of breast area and its intersection.

However, as seen in Figure 9b, there is not a clear definition of the desired area, so using the information of pixels provided in the previous step, an approximation using two second-degree polynomials of the form $y=a_{0}+a_{1}x+a_{2}x^{2}$, estimated using a linear regression, can be used to infer the functions that complete the way below the breast areas, as shown in Figure 10. In such an equation, *y* denotes the dependent variable of *x*; meanwhile, $a_{0}$, $a_{1}$ and $a_{2}$ are the function coefficients estimated with the linear regression. A sweep on the x-axis allows one to determine the pixels corresponding to each function based on the behavior of the y-coordinate. The intersection of both estimated lines with the second-degree polynomials can be used as a control point to separate the left and right breast.

**Figure 11:**
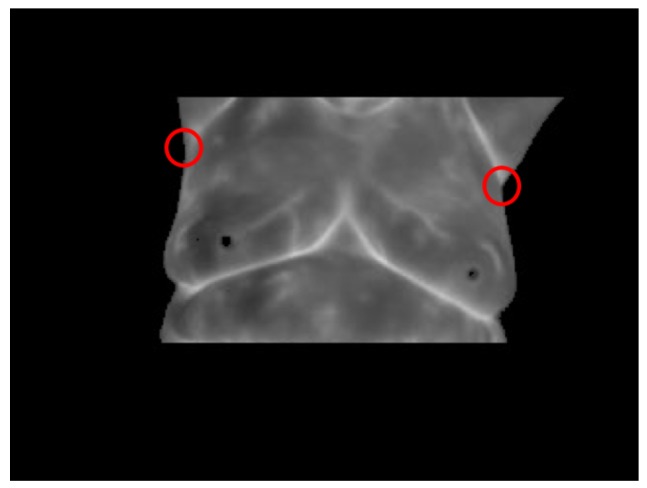
Armpit points with the highest slope variation.

Finally, to achieve a fully-automatic segmentation of the breast area, it is necessary to establish an appropriate way to find the upper limit of it. In the different tests, it was observed that, because of the proposed protocol for the thermograms’ acquisition, a natural slope in the armpits was always present. This characteristic can be seen in Figure 11. Therefore, from the binary image resulting from Otsu’s method and estimating the contours using a morphological gradient, it was necessary to find the points with the most significant variation of slope located above the lower limit of the breast area detected in the previous step both from the right and left armpit. Using a numerical differentiation over the contour lines of the armpits, considering the two-point formula to compute the slope of a nearby secant f(x+h)−f(x−h)/2h, with h=10, the slope change gradient along the armpit lines was determined. In the two-point formula, *f* denotes the function line of the armpit, *x* is the current point where the slope is being estimated and *h* is the step forward and backward, in pixels, considered to estimate the slope. When the points with the larger slope variation on both lateral sides of the patient silhouette are found, the next step consists of drawing a line between them and the intersection point of the two polynomial functions estimated in the previous step and shown in Figure 10 to achieve a segmentation of the desired area.

**Figure 12:**
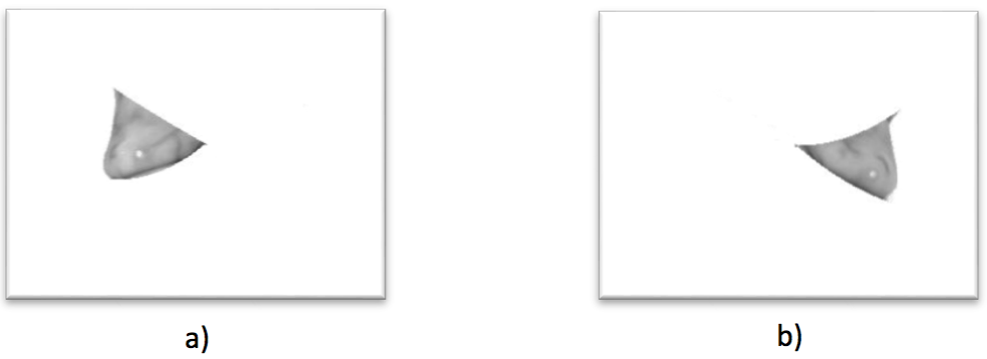
Segmented breast thermograms: (**a**) left; and (**b**) right breast.

The results of the automatic segmentation proposed can be seen in Figure 12. This segmentation automatically separates the desired regions of interest so they can be treated independently to analyze the temperature in the right, Figure 12a, and left, Figure 12b, breast.

### 2.4. Evaluation

It is stated that a difference of temperature in the corresponding symmetry region in the human body can be an indicative of health problems. A difference of 1 °C can be indicative enough to establish a normal or an abnormal condition. Therefore, it is necessary to perform an analysis to compare the temperatures present in the segmented right and left breast. To perform such an analysis, first, it is necessary to transform the gray scale intensity levels of the acquired thermogram to a thermal scale; this is a matrix of temperatures. This can be achieved with Equation (3), where $T_{r}$ denotes the real temperature of the pixel, $T_{min}$ and $T_{max}$ are the minimal and maximum value of temperature from the scene registered by the FLIR software, $T_{gray}$ is the gray value of intensity of the pixel and $T_{vgm}$ is maximum intensity gray level present in the thermogram.

(3) $$T_{r}=T_{min}+T_{gray}/T_{vgm}*\left(T_{max}-T_{min}\right)$$

Next, an average of the temperature of the segmented areas is estimated, ${\bar{T}}_{right}$ and ${\bar{T}}_{left}$ for right and left mean breast temperatures, respectively, which allows one to perform a comparison between the sides. In the same way, two cases can be established, for a normal case where the presence of cancer and angiogenesis can be discarded, the condition shown in Equation (4) should be fulfilled.

(4) $$|{\bar{T}}_{right}-{\bar{T}}_{left}|<1{}^{\circ}C$$

For the second case, where the difference in temperatures between the right and left breast result in being equal or higher than 1 °C, as noticed in Equation (5), the risk of cancer or angiogenesis is highly possible, suggesting an asymmetrical behavior of temperatures. As was mentioned before, the cancer cells present an accelerated metabolism, causing a rise of temperature in the tumor region.

(5) $$|{\bar{T}}_{right}-{\bar{T}}_{left}|\geq 1{}^{\circ}C$$

In the case when a difference was found, an analysis of the breast with the highest temperature is performed. This analysis searches the hottest point or region in the breast to delimit it using a morphological segmentation tool.

### 2.5. Diagnosis

**Figure 13:**
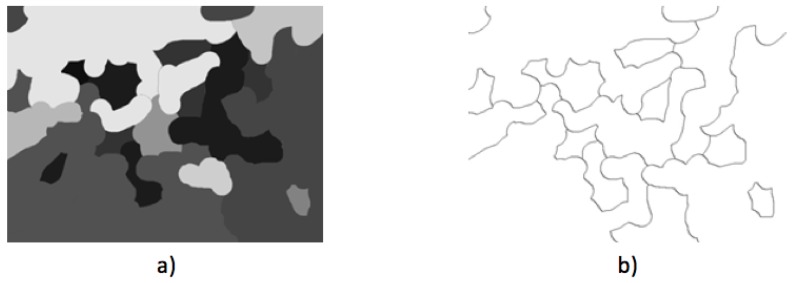
Example of watershed segmentation: (**a**) synthetic test image; and (**b**) the resulting segmented image.

If the evaluation analysis yields a difference of temperatures less than 1 °C, then the systems deliver the user a message “no problem detected” and a green indicator denoting that no problem was found. For the second case, which is when the difference of temperatures is equal or higher than 1 °C, a message with the label “problem detected” is shown followed by a red indicator; however, another step is performed internally. The objective of this additional step is to spot the zones with the highest temperature, which can be used by the experts to find the location of a tumor. To achieve this, a segmentation of the side breast with the highest mean temperature is performed using the watershed technique, which is a powerful tool used in mathematical morphology [53]. The watershed operator considers the image as a topographic surface where the gray level of a certain pixel indicates its altitude. A flood is simulated over the image from the regional minima. When the water coming from the neighbor minima values gets in touch, a new watershed line is formed [54]. An example of the watershed operator over a synthetic image can be seen in Figure 13. For this case, Figure 13a represents a thermogram with multiple temperature values denoted in different gray intensity levels, and Figure 13b is the result of the flood process after the minima estimation where the different regions are now separated by the formation of the watershed lines.

The application of the watershed operator leads to the possibility to diagnose breast cancer, but also angiogenesis cases; such cases are developed in the next paragraphs.

### 2.6. Angiogenesis

**Figure 14:**
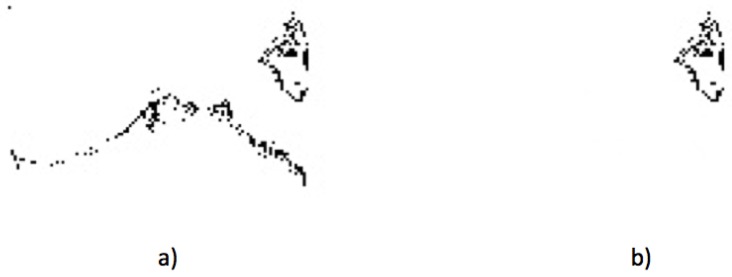
Angiogenesis detection example: (**a**) the hottest regions detected in a full thermogram; and (**b**) an isolated pattern indicating angiogenesis.

Angiogenesis is a state in the blood vessels that is present in the early stages of the development of breast cancer. Because of this, an additional case of detection implies the use of the segmented thermograms and the use of the watershed tool to find regions with a behavior that is clearly different from that shown in the cancer case. In Figure 14, an example case of angiogenesis detection is shown. The processing work is similar; the watershed tool can be used to find multiple hot regions, as can be seen in Figure 14a; however, this time, the temperature pattern is very peculiar, and it can be associated with an abnormality in the sanguineous vessels, as shown in Figure 14b.

### 2.7. Cancer

**Figure 15:**
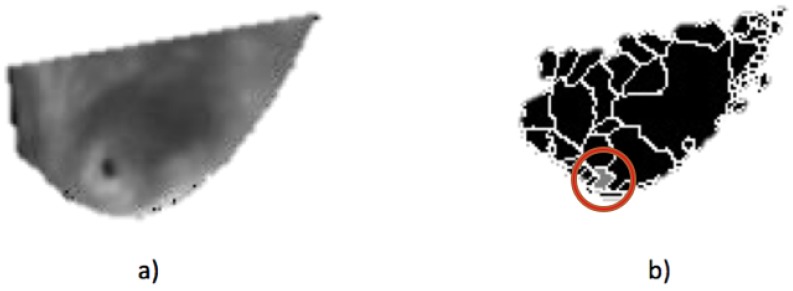
Breast cancer detection example: (**a**) segmented right breast thermogram; and (**b**) the result of watershed segmentation with the hottest spot detected in gray.

For the breast cancer case, the process shown in Figure 15 is followed. When the evaluation of the thermal matrix establishes that one side presents a higher temperature than the other side, the final diagnosis is centered on such a breast side, as shown in Figure 15a; the result of applying watershed segmentation is presented in Figure 15b, in which the regions with different temperatures are now separated, easing finding the hottest point denoted in a gray color surrounded by a red circle for visualization purposes. With the hottest temperature point located in the breast thermogram, this information can be presented to both the patient and an expert to continue with a diagnosis bearing in mind this knowledge.

## 3. Results and Discussion

### 3.1. Validation

In order to validate the proposed method, images from the Database for Mastology Research (DMR) were used [41,55]. Such a database includes thermograms that are classified as healthy or sick, with diagnoses that were obtained from mammography of biopsy studies. Images from static and dynamic acquisition protocols are included. Specifically, for the static protocol, images are acquired at 1 m and with a thermal stabilization time of 10 to 15 min. Such time corresponds to a difference of 60% with the proposed stabilization time for this job. From the image set classified as healthy, 37 thermograms were selected randomly; meanwhile, all 42 classified as sick were used in the validation. Our system achieves an 80.95% match for healthy cases and a 78.26% for the sick cases, compared with the diagnosis provided in the DMR dataset; however, also, it is important to establish that for 44.3% of the analyzed images, our system was unable to provide a result due to an incorrect breast segmentation. The results of such an analysis can be seen in Table 2.

Therefore, the TPR (true positive rate) or sensitivity, after classifying correctly as sick 18 out of 23 sick cases, is 0.7826; the FPR (false positive rate) of the test, in which four healthy cases of 21 were classified as sick, is 0.1904; and the false negative rate (FNR), after classifying five sick cases as healthy, is equal to 0.2173. Finally, the specificity of the test is 0.8095 [56]. Such a situation can be attributed to the mentioned differences in the thermal stabilization times and, in general, to the capture protocol. As was mentioned, the proposed protocol is fundamental for our system to ease the detection of the lower breast limits. It is important, therefore, that the stabilization time is equal to that reported in Figure 4, to ensure a correct operation of the proposed segmentation method. When the proposed protocol for the thermograms acquisition is followed, both the results of segmentation and the percentage of classification match deliver better results, as can be seen in Table 3.

For the proposed segmentation method and when the protocol for image capture has been followed, there is a segmentation efficiency of 90.30%, which is obtained from the success of segmentation and delivery of the result after the first thermogram capture. For the remaining cases, second or third captures were required to deliver a result, and for 1.98% of the 454 tests, representing nine patients (seven healthy and two sick), the system was unable to deliver a result. Such a situation can be attributed to women with very complex body constitutions or cases where the patients could not keep their arms in the nape because of physical impediments. The TPR (sensitivity), in this case, reaches 0.8684 after classifying correctly as sick 33 of 38 cases. The FPR results in 0.1056, after classifying as sick 43 of 407 healthy cases; meanwhile, the FNR now is equal to 0.1315, after delivering a healthy status in five of 38 sick cases. The specificity of the system after the clinical trial reaches 0.8943.

### 3.2. Test

In order to test the proposed methodology, as well as the proposed acquisition protocol, a series of analyses were performed in a group of 454 voluntary women. This group included women of different ages and health conditions. Many women participated in this study whose health conditions ranged from no detected problems to women with angiogenesis or cancer in different stages already diagnosed by an oncologist; pregnant and breastfeeding women participated, as well, in the study. Each woman who participated in the test signed a responsive sheet that specifies the null effects of infrared thermography, and it was agreed that the images acquired would be subject to scientific use while preserving the privacy of their data. Finally, the tests were carried out following the guidelines recommended by the ethics committee of the Autonomous University of Queretaro. This study was performed in the area of Integral Health Nursing in the Faculty of Nursing of the same school. The invitation to participate in the study was conducted openly; those who accepted signed a letter of informed consent. The study was conducted with the support of a group of nurses of the Autonomous University of Queretaro. In this way, the requirements of standard medical procedures were followed.

### 3.3. Cases with No Problems Detected

The next cases are representative of a series of performed analyses in which no problems were found. The examples shown include women with two different body constitutions, robust and slim, to show how the proposed methodology works correctly in both cases.

**Figure 16:**
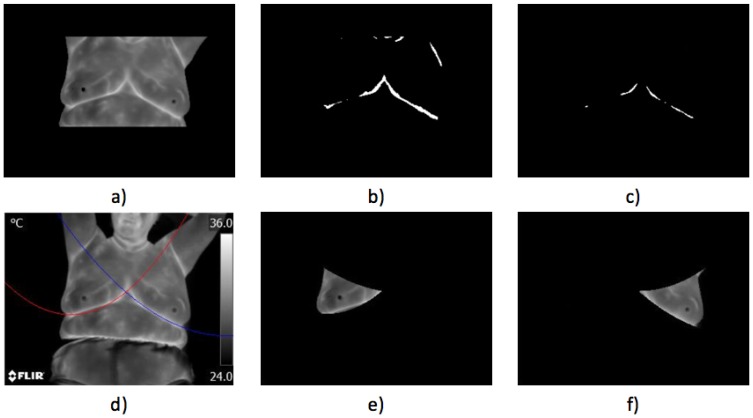
Case 1: (**a**) original acquired thermogram; (**b**,**c**) lower limit detection; (**d**) second degree polynomials; (**e**) right; and (**f**) left segmented breasts.

**Figure 17:**
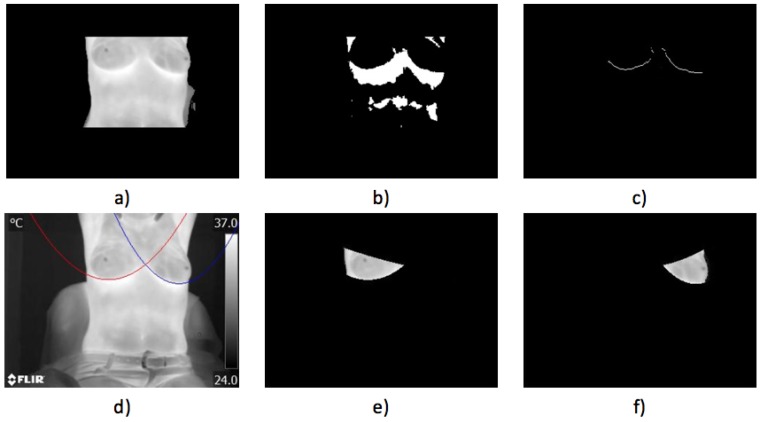
Case 2: (**a**) original acquired thermogram; (**b**,**c**) lower limit detection; (**d**) second degree polynomials; (**e**) right; and (**f**) left segmented breasts.

- Case 1, healthy:
  The first case illustrated in Figure 16 implies a woman with a robust constitution. The acquired thermogram can be seen in Figure 16a; the estimation of the inferior limits of the breast area can be seen in Figure 16b–d. The segmented right breast is shown in Figure 16e; meanwhile, the left is depicted in Figure 16f.
  The average temperatures of the segmented breast sides are shown in Table 4. The difference of temperatures is less than 1 °C, establishing that no problem was found. Maximum, minimum and standard deviation values are also depicted. The differences, in the three cases, also are less than 1 °C between the left and right sides.
- Case 2, healthy,
  In Figure 17, the proposed methodology is applied to a woman with a thin constitution. In the acquired thermogram shown in Figure 17a, it is possible to see how a small region corresponding to the seat was segmented together with the patient body by Otsu’s method; however, this does not mean any problem for the finding of inferior limits exhibited in Figure 17a–d. Although at this time, the armpit slope is lighter than the previous case, the proposed segmentation results in being robust enough to separate the right and left breast zones, as can be seen in Figure 17e,f.
  Finally, the average temperatures present in the right and left breast side are shown in Table 5. Again, the difference of temperatures is less than 1 °C, with a result of no problem detected. This time, the difference between the maximum values is greater than 2 °C and could be associated with the segmentation result shown in Figure 17e, which also includes the side of the breast.

### 3.4. Cases with Problems Detected

The following cases involve women where cancer or early stages of cancer were found. Again, different body constitutions are presented to show the correct working of the proposed methodology.

**Figure 18:**
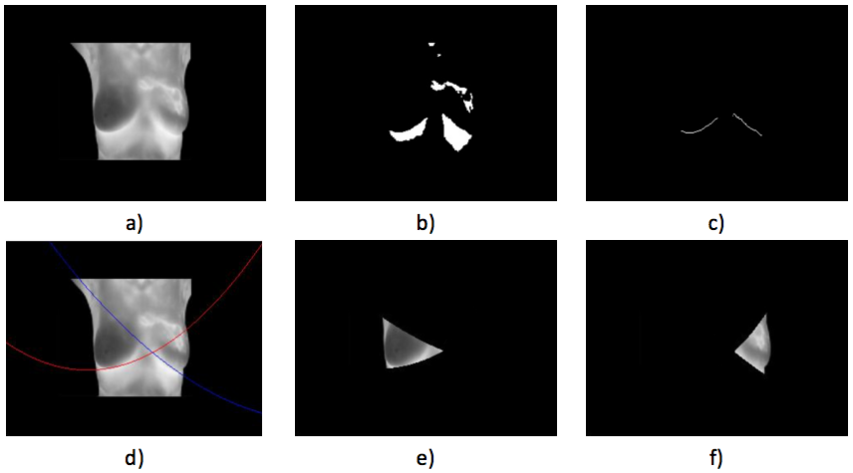
Case 3: (**a**) original acquired thermogram; (**b**,**c**) lower limit detection; (**d**) second degree polynomials; (**e**) right; and (**f**) left segmented breasts.

**Figure 19:**
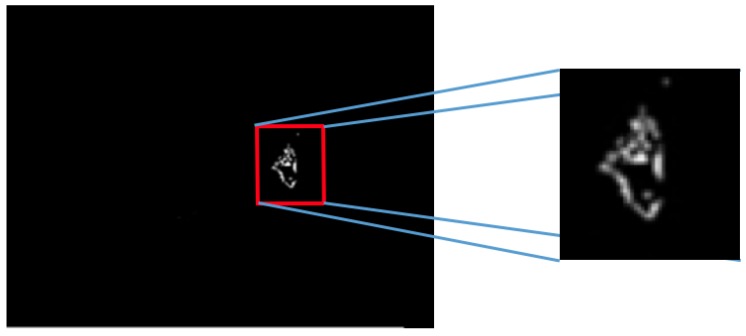
Hottest region detected for Case 3 resembling an angiogenesis case.

**Figure 20:**
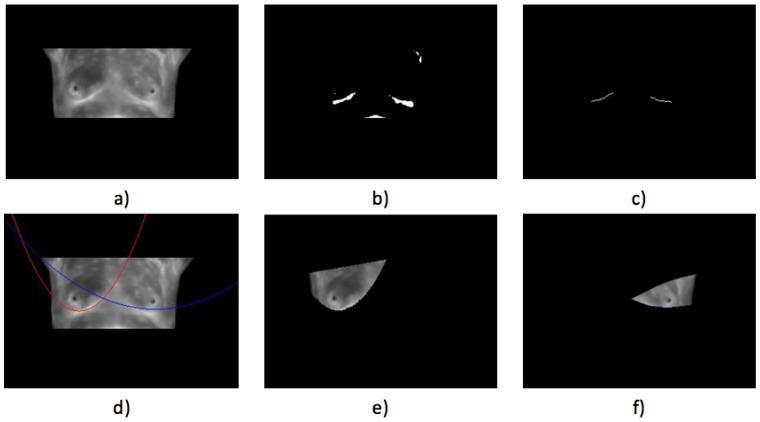
Case 4: (**a**) original acquired thermogram; (**b**,**c**) lower limit detection; (**d**) second degree polynomials; (**e**) right; and (**f**) left segmented breasts.

**Figure 21:**
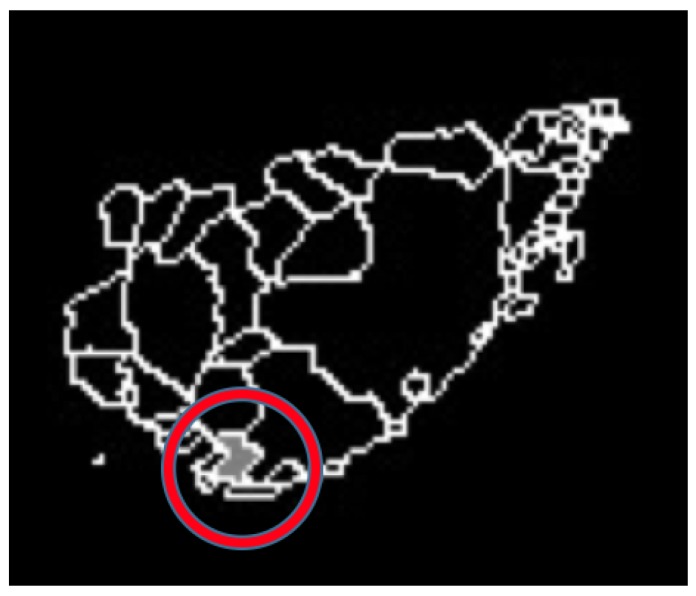
Hottest point detected in the right breast for Case 4 indicating the location of a cancer tumor.

- Case 3, angiogenesis:
  The acquired histogram is shown in Figure 18a, and as in the previous cases, the pre-processing necessary to segment the breast regions can be followed step by step in Figure 18b–d. The segmented right and left breast are shown in Figure 18e,f, respectively. As in the previous case, a noticeable difference of temperatures can be seen in the thermogram in Figure 18a, with a lighter zone in the upper region of the left breast of the patient. This comparison is easier to achieve looking at Figure 18e,f.
  In Table 6 can be seen the estimation of the average difference of temperatures, which result in being 1.81 °C, with the left breast resulting in being hotter than the right breast, as expected. This is a clear indication of a detected problem, and now, the search for the hottest regions in the left breast is required. For this case, the difference of the maximum value and the standard deviation between the left and right sides is quite significant. In Figure 18, it is clear that one breast is lighter (or hotter) than the other.
  Watershed segmentation result can be seen in Figure 19. This time the hottest region is surrounding a cold zone and the resulting segmented shape can be associated with the one that blood vessels present resulting in an angiogenesis case. As mentioned earlier angiogenesis can be seen in early stages of breast cancer and subsequent medical exams are required, having the point of interest spotted in order to help the experts to correctly treat the patient.
- Case 4, breast cancer:
  The last case can be seen in Figure 19. The acquired thermogram is shown in Figure 20a. The detection of the breast inferior limits can be seen in Figure 20b–d; meanwhile, the segmented right and left breast are depicted in Figure 20e,f. In the previous two cases, a more uniform temperature distribution was noticeable; in this case, however, multiple regions with high temperatures can be seen in Figure 20a in lighter shades of gray getting close to white. Although a qualitative analysis is not the objective of this paper, such information can be used to infer the existence of a problem that must be analyzed.
  Posterior to the breast segmentation, the evaluation process delivers the results shown in Table 7. As can be seen, the average temperature difference is 1.92 °C, which is slightly superior to that presented in Case 3. The maximum value and standard deviation present the highest difference of the four cases analyzed, reaching a value of 8.53 °C for the first one and 2.46 °C for the second one.
  With the numeric result establishing a difference of temperatures between right and left breast higher than 1 °C, the additional step, which implies spotting the location of the hottest region, is performed. Watershed segmentation applied to the right breast, which results in being the one with the highest temperature average, is shown in Figure 21, with the region with the highest temperature denoted in gray and indicated within a red circle; such a region is located in the lower zone of the breast, and the expert is recommended to be aware of it.

## 4. Conclusions

In this paper, a tool whose purpose is to help in the detection of breast cancer using a thermographic camera as a sensor was presented. IR thermography is a powerful tool that results in being non-invasive and non-intrusive, which eases the process of analysis, providing safety and comfort to the patients, advantages that this work provides. The proposed methodology can be used in women of different ages and health conditions without representing any kind of risk. Three main challenges in breast cancer detection using IR sensing technology were faced in this research. First, the proposed tool is based on an automatic segmentation methodology founded upon the use of image processing algorithms, and the use of a geometrical approach to achieve the left and right breast segmentation resulted in being robust enough to achieve this, even when the lower limit of the breast was not very defined or when, in the search of the upper limit, the slope in the armpits was not very marked. Second, in order to achieve a good behavior of the automatic segmentation methodology, a protocol for image acquisition was presented, establishing a target time to reach a thermal stabilization in order to minimize the possibility to get bad measurements. Thermal stabilization helped to decrease the operating range of the IR sensor, which resulted in being useful when the grayscale thermogram was converted to the thermal matrix reaching the resolution aspect, being able to detect smaller changes of temperature. Third, the evaluation of thermograms was complemented with a watershed segmentation tool that made it possible to spot regions with higher temperatures in the hottest breast side analyzing the thermal matrix of it. This information resulted in being important to the experts because it attracts attention to that region. Finally, the tool presented in this paper was validated using the Database for Mastology Research, and although the sensitivity and specificity reached were 0.7826 and 0.8095, respectively, it is important to mention that such a database was acquired considering a thermal stabilization of 10 min, which represents 40% of the time proposed for this job, causing only 55.7% of the images being able to be classified. The proposed methodology also was tested in a group of 454 voluntary women. The cases shown in this paper were verified by an oncologist and women counted with a previous diagnosis, obtained by traditional methods, of healthy or cancer in different stages. In this clinical trial, following the proposed image acquisition protocol, the sensitivity and specificity obtained increased to 0.8684 and 0.8943, respectively, and a segmentation efficiency of 90.30% was reached. Although the present work uses an industrial-grade IR thermographic sensor, it can be easily modified to work with other commercial options to ease its implementation in marginal regions in developing countries.

## Figures and Tables

**Table 1:** Characteristics of the IR thermographic sensor FLIR A-300.

Characteristic	Specification
Sensibility	0.05 °C
Spatial resolution (IFOV)	1.36 mrad
Thermogram resolution	320 × 240 px
Sensor speed	25 us
Thermogram format	JPEG
Communication	Ethernet
Emissivity correction	Yes, from 0.1 to 1.0
Focus	Automatic
Zoom	Digital, up to 8×

**Table 2:** Comparison of the results using the Database for Mastology Research (DMR).

Cases (Total)	Healthy	Sick	Unknown
Healthy (37)	17	4	16
Sick (42)	5	18	19
Total (79)	22	22	35

**Table 3:** Validation using our clinical trial.

Cases (Total)	Healthy	Sick	Unknown
Healthy (414)	364	43	7
Sick (40)	5	33	2
Total (454)	369	76	9

**Table 4:** Statistical values of the temperatures of the segmented left and right breast areas and difference estimation for Case 1.

Temperature	Left	Right	Difference
Average	26.43 °C	25.70 °C	0.73 °C
Max	35.56 °C	36.5 °C	0.94 °C
Min	24.04 °C	24.04 °C	0 °C
SD	3.34 °C	3.61 °C	0.27 °C

**Table 5:** Statistical values of the temperatures of the segmented left and right breast areas and difference estimation for Case 2.

Temperature	Left	Right	Difference
Average	29.07 °C	29.65 °C	0.58 °C
Max	36.45 °C	34.29 °C	2.16 °C
Min	24.04 °C	24.04 °C	0 °C
SD	3.60 °C	2.98 °C	0.62 °C

**Table 6:** Statistical values of the temperatures of the segmented left and right breast areas and difference estimation for Case 4.

Temperature	Left	Right	Difference
Average	30.1 °C	28.29 °C	1.81 °C
Max	36.45 °C	33.60 °C	2.85 °C
Min	24.04 °C	24.04 °C	0 °C
SD	3.60 °C	2.78 °C	0.82 °C

**Table 7:** Statistical values of the temperatures of the segmented left and right breast areas and difference estimation for Case 4.

Temperature	Left	Right	Difference
Average	26.91 °C	28.84 °C	1.92 °C
Max	27.82 °C	36.35 °C	8.53 °C
Min	24.04 °C	24.04 °C	0 °C
SD	1.11 °C	3.57 °C	2.46 °C
